# Prevalence of Streptococcus pyogenes as an oropharynx colonizer in children attending daycare: a comparative study of different regions in Brazil

**DOI:** 10.1016/S1808-8694(15)31013-2

**Published:** 2015-10-19

**Authors:** Fernando Mirage Jardim Vieira, Cláudia Regina Figueiredo, Maria Claudia Soares, Lily Yin Weckx, Odimara Santos, Gleice Magalhães, Patrícia Orlandi, Luc Louis Maurice Weckx, Shirley Pignatari

**Affiliations:** a Graduated in Medicine from the UNIFESP-EPM. Resident Doctor; b PhD from the UNIFESP-EPM, Associated Doctor at UNIFESP-EPM.; c PhD from the UNIFESP-EPM, Associated Doctor at UNIFESP-EPM.; d PhD from the UNIFESP-EPM, Associated Professor at UNIFESP-EPM.; e Graduated in Biology, Microbiologist, Pediatric Otorhinolaryngology Discipline at UNIFESP-EPM.; f Graduated in Biology, Post-Graduate Student at Rondônia Federal University; g PhD in Biology from the University of São Paulo, Biologist at IPEPATRO.; h Professor at UNIFESP-EPM, Head of Otorhinolaryngology and Head and Neck Surgery Department at UNIFESP-EPM.; i PhD in Otorhinolaryngology at UNIFESP/EPM, Chief of Pediatric Otorhinolaryngology Discipline at UNIFESP/EPM. Pediatric Otorhynolaringology Discipline at São Paulo Federal University - Escola Paulista de Medicina - Rua dos Otonis 684 Vila Clementino São Paulo SP 04025-001.

**Keywords:** child day care centers, oropharynx, streptococcus pyogenes

## Abstract

Thirty percent of acute pharyngotonsillitis is caused by Streptococcus pyogenes, which increased the risk of glomerulonephritis and rheumatic fever. Children attending daycare centers have a higher incidence of these infections.

**Aim:**

to identify and compare the prevalence of Streptococcus pyogenes in the oropharynx of children who are enrolled and who are not enrolled in daycare centers in different regions of Brazil.

**Materials and Methods:**

A prospective study of two hundred children from Sao Paulo/SP and Porto Velho/RO. Children from each city were divided into two groups: those attending, and those not attending daycare centers. Swabs of the oropharynx were taken for bacteriological culture and identification.

**Results:**

The prevalence of Streptococcus pyogenes in the São Paulo groups were 8% and 2% for daycare and control groups, which was statistically significant (p=0.02). The prevalence in children from Porto Velho/RO was 24% and 16% for daycare and control groups, which was statistically significant (p=0.015). Statistical analysis also showed a significant difference between the corresponding groups in the two locations (p<0.01).

**Conclusion:**

These results show that daycare attendance is a risk factor for oropharyngeal streptococcal colonization; this was seen in different populations, but was statistically significance in only one of the two samples.

## INTRODUCTION

Acute tonsillitis is extremely frequent in the pediatric population, and although palatine tonsils show bacterial colonization by polymicrobial flora, including anaerobius and aerobius (e.g. S. mutans and S. pyogenes, Staphylococcus aureus, Moraxella catarrhalis, Haemophilus influenzae, Prevotella sp, Bacteróides fragilis and Fusobacterium sp^1^, ^2^, ^3^) the main concern among children's healthcare professionals still is Streptococcus pyogenes - a Lancefield Group A beta- hemolytic microorganism (SBHGA). About 30% to 40% of acute pharyngotonsillitis are of streptococci etiology^4^, ^5^, ^6^, and the importance of this fact is due to the possibility of late non-suppurative complications caused by this pathogenic agent, such as for example, acute diffuse glomerulonephritis and rheumatic fever, which represents 90% of surgery indications for heart valve replacement in children in Brasil.^7^

Besides, specially on the pediatric population, acute tonsillitis represents a large source of social inconveniences, such as missing days of school, the need to use antimicrobial drugs repeatedly, and the potential risk of suppurative complications.^8^

Most studies have focused in searching alterations in the oropharynx flora of children with persistent tonsillitis. Most of these studies have shown a higher prevalence of organisms producing beta-lactamase and also a higher variety of organisms in this population. Streptococcus pyogenes prevalence has been higher among these children, when compared to that in children who do not present persistent tonsillitis. However, a statistical difference was not found in the studies analyzed.^1^, ^2^, ^9^, ^10^, ^11^, ^12^, ^13^

Due to the great importance that streptococci infection epidemiology has in our country, the efforts to better understand its risk factors are justified.

During the last two decades, a socio-economic phenomenon has been observed, with the significant increase of women working outside the home, and as a consequence, an increase in the number of pre-school age children attending nursery schools.

Among the children attending nursery schools (those institutionalized), the incidence of upper airways infections is 2 to 18 times higher than in children the same age, who do not attend nursery schools. Besides, infections in children who attend nursery schools are usually more severe, requiring antibiotic therapy for a period up to four times longer than those children who do not go to nursery schools ^8^.

This study aims to identify and compare the prevalence of Streptococcus pyogenes in the oropharynx microbial flora of healthy children attending nursery schools and in non-institutionalized children in different regions of the country.

## CASE STUDY AND METHOD

Two hundred children took part in this study. After obtaining the free and clear authorization from parents or guardian, we applied a questionnaire addressing previous ear, nose and throat infections. This study was submitted for assessment and it was approved by the Research Ethics Committee at UNIFESP-EPM, protocol number 1398/03.

The children were divided into 4 groups:

- Group I: 50 institutionalized children from “Mãe do Salvador” nursery school, located in São Paulo/SP; 23 (47%) were male and 27 (53%) female, with a minimum age of three months old, a maximum age of three years old and an average age of one year and 10 months.
- Group II: 50 non- institutionalized children at the outpatient Children's Health Center at Vila Mariana in São Paulo/SP; 27 (54%) were male and 23 (46%) female, with ages ranging from six months to 3 years, mean age of one year and 11 months.
- Group III: 50 institutionalized children from “Trem da Alegria” nursery school located in Porto Velho/RO, 28 (56%) were male and 22 (44%) female, with ages ranging from one to eight years, mean age of four years and three months.
- Group IV: 50 non-institutionalized children from Ana Adelaide Pediatric Outpatient Center, in Porto Velho/RO, 27 (54%) were male and 23 (46%) female, with ages ranging from one to eight years, mean age of four years and three months.

Inclusion criteria:

• Healthy children not older than 10 years.
• Normal ear, nose and throat exam

Exclusion criteria:

• Use of antibiotic therapy in the last 15 days
• Previous tonsillectomy
• Eating within the two hours before taking the sample
• Congenital or acquired immunedeficiencies
• History of persistent tonsillitis (3 or more events in 6 months or 4 events in 1 year)

In the city of São Paulo, the samples were taken during the months of June and July which corresponds to the dry winter in the State of São Paulo. Whereas in Port Velho, the samples were taken during the months of September and October, hot and humid periods which precedes the rainiest season in the region.

The oropharynx material was collected using direct view with alginate swab (LaborClin®). The material was immediately placed on culture medium for aerobius (chocolate-agar/blood-agar) by a microbiologist in all samples.

The samples were taken following the microbiologic protocol described as follows. Culture dishes were immediately taken to a lab and incubated in a bacteriology furnace at 36ºC with environment enriched in CO2 for 24 hours.

After 24 hours, the plates were assessed regarding the colonies growth and morphology. Those which did not show bacterial growth were incubated again for 24 more hours.

The identification of bacteria was done according to the manual techniques established by the Washington Medical School in the “Manual of Clinical Microbiology” (Handbook of Clinical Microbiology).^14^

Samples taken in Porto Velho/RO were submitted to culture and analysis at Ipepatro (Tropical Diseases Research Institute), a federal research institution linked to the Ministry of Health, following the same protocol used in São Paulo/SP, at the microbiology lab of Pediatric Otorhinolaryngology Discipline at UNIFESP/EPM.

### Statistical Analysis

For the analysis of the possible associations among the groups on the presence or absence of Streptococcus pyogenes, the chi-squared test (c 2) was used. For the association tables, Yates’ correction was used, whenever necessary. When Cochrane's restrictions were present, the chi-squared test was replaced by Fisher's Exact Test.15 In all cases, rejection level for the null hypothesis was always fixated in a value lower or equal to 0.05 (5%).

## RESULTS

The two hundred children showed bacterial growth in culture. Streptococcus pyogenes was present in 8% of cultures in children from the nursery school group and in 2% in the control group in the city of São Paulo, showing a significant statistical difference between both groups (p=0.02). The same microorganism was present in 24% of the samples in the nursery school group and in 16% of the samples in the control group in the city of Porto Velho, without showing a significant statistical difference between the samples (p = 0.18).

The comparison between the control groups of the different regions shows a higher prevalence of streptococcus in the Porto Velho population, with a significant statistical difference (p<0.01). The same happens when comparing the groups of children attending nursery school (I and II), and this difference also showed statistical significance (p<0.01).

## DISCUSSION

Acute pharyngotonsillitis is an infectious disease, very common in the pediatric population, and besides the clinical condition, it is normally associated with a worsening of general condition, high fever, odynophagia and important discomfort for the child, streptococci pharyngotonsillitis could also lead to serious non-infectious complications, such as rheumatic fever and post-streptococci glomerulonephritis.^8^

**Figure 1:**
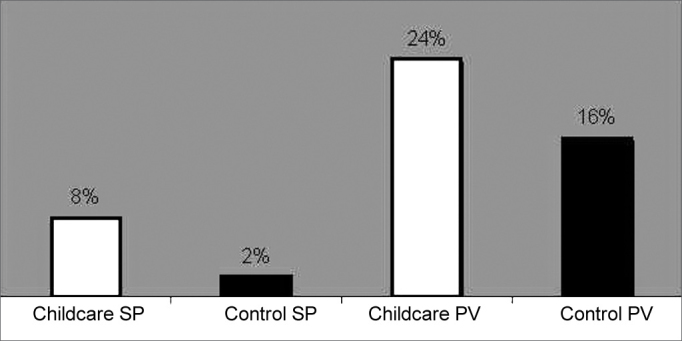
Prevalence S. pyogenes colonization in the four groups studied - Significant statistical difference in the groups from São Paulo/SP.

**Figure 2:**
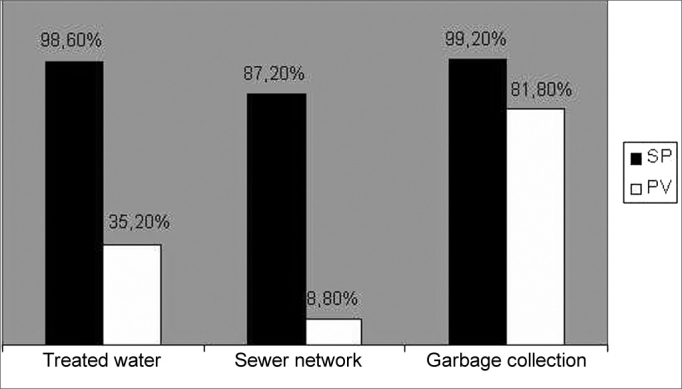
Comparative chart of basic sanitary conditions at households in São Paulo/SP and Porto Velho/RO - Source IBGE (Brazilian Institute of Geography and Statistics)

Although literature is not very rich on this subject, generally speaking, it is believed that children attending nursery schools as a routine, show a higher risk of having more severe and longer pharyngotonsillitis, and they frequently demand continuous and more extended treatments with antibiotics.^8^

On this study, authors made a comparative analysis of SBGHA presence in the oropharynx of both institutionalized and non-institutionalized children. Oropharynx material was collected outside the acute episode, and children with persistent tonsillitis history were excluded - in order to avoid individual factors that may alter the oropharynx colonization.

Children in São Paulo, both those attending nursery schools and the non-institutionalized ones, show similar socio-economic characteristics and although they are from institutions situated in rich areas of the city of São Paulo, the population included in the study does not correspond to the same socio-economic pattern of the residents of these areas, since they attend these institutions because their parents work on this region of the city, and they are mainly characterized as low economic class.

Similarly, children in Porto Velho belonged to population groups characterized as medium and medium low economic classes, with the control group (non-institutionalized children) being from families with a slightly lower average family income, most of them being low class.

In our study, SBHGA was detected in the four groups, with predominance in the group of children attending nursery schools when compared to the control group of the city of São Paulo. It was also clearly seen a higher prevalence of the microorganism in Porto Velho/RO groups when compared to those from São Paulo/SP.

The results found in the children from São Paulo reproduce international series which found SBHGA prevalence as colonizer in 5% to 10% of the children studied.^2^, ^10^, ^11^

Nevertheless, the results of Porto Velho populations show a higher prevalence, greater than the results found in all the analyzed publications.

The cities of São Paulo and Porto Velho have their own features and they differ greatly on climate and socio-economic conditions. São Paulo has a population of 10 million inhabitants and it is situated on the Southeast, 70km from the coast, it has mesothermic climate with up to three months of dry season per year, with an average temperature of 15 to 20°C and a significantly lower humidity than Porto Velho.^16^

The city of Porto Velho is approximately 3.600km away from São Paulo. It is situated on the Northern region of the country and it is the capital city of the State of Rondônia. It is characterized by being a medium size city with a population of 380.000 inhabitants. In comparison to São Paulo, the population in Porto Velho has a lower income per inhabitant and it faces significantly worse sanitary conditions. It has a warm and humid equatorial climate with average temperatures above 24°C and a high rain index during the whole year. It is surrounded by the Amazon forest and it is situated near the Rio Madeira (River Madeira), next to the Bolivian border.^16^

The samples taken in both cities included in the research study were made in different seasons and the isolation and identification of microorganisms were done in different labs, however using the same standardization. It should also be noticed that the average age of children in São Paulo was one year and eleven months old, whereas the average age of children in Porto Velho was four years and three months old, an age group that normally has more colonization by SBHGA, due to a higher social contact. We consider that these differences may represent limiting factors for a conclusive comparison between both populations. However, it should be noticed that despite these differences, the results show a trend to higher colonization by SBHGA in children attending nursery schools in both samples.

Attending nursery schools is a risk factor established for persistent tonsillitis, and this study points to the fact that it should also be treated as a risk factor of colonization of oropharynx by the pathogenic microorganism. SBGHA transmission is done through interpersonal contamination by upper airway secretion drops, facilitated by the gathering and interpersonal contact observed in nursery schools.

Unfortunately, the number of children attending nursery schools is increasing, since it is a need for many families. Despite the difficulty of dealing with this likely risk factor, the otorhinolaryngologist should be aware of the high incidence of this streptococci infection in this group of patients to better guide his/her therapeutic strategy.

Despite the different sample taking conditions between the two cities, the main difference of results raises a doubt on the epidemiological data of Brazilian regions far away from the main research centers. These findings question the capacity that studies done in São Paulo have to represent, in a precise manner, the reality of the whole country. We understand that from these remarks, it is justified to carry out multi-centered studies to determine SBHGA prevalence in our country.

The North region in our country is extremely rich in natural resources and it is increasingly attracting more international interests. However, it is still very far from the attention received by the population living in the large Brazilian cities. Without economic and social development, its population lives a very different reality from those living in the more developed areas of the country, as we managed to exemplify on this study. The need to develop and to integrate the most distant areas in Brazil is evident, and it is a challenge the authorities of this country face for the future.

## CONCLUSION

The results of this study suggest that nursery schools may represent a risk factor for colonization of oropharynx by Streptococcus pyogenes, a fact observed in a statistically significant manner in the groups from the city of São Paulo. The results also suggest that the prevalence and colonization of the oropharynx in children by this bacterium is higher in the city of Porto Velho / RO, when compared to the city of São Paulo / SP.
